# Caregiver perceptions regarding the measurement of level and quality of care in Alzheimer’s disease

**DOI:** 10.1186/s12912-015-0104-8

**Published:** 2015-10-24

**Authors:** Afeez Abiola Hazzan, Jenny Ploeg, Harry Shannon, Parminder Raina, Mark Oremus

**Affiliations:** Department of Medicine, McMaster University, 1280 Main Street West, Hamilton, ON Canada, L8S 4K1; Department of Clinical Epidemiology and Biostatistics, McMaster University, 1280 Main Street West, Hamilton, ON Canada, L8S 4K1; School of Nursing, McMaster University, 1280 Main Street West, Hamilton, ON Canada, L8S 4K1; School of Public Health and Health Sciences, University of Waterloo, 200 University Avenue West, Waterloo, ON Canada, N2L 3G1

**Keywords:** Alzheimer’s disease, Primary caregivers, Level of care, Quality of care, Qualitative content analysis

## Abstract

**Background:**

Primary informal caregivers play a critical role in the care and support of persons with Alzheimer’s disease (AD). A recent systematic review found little existing research into whether caregiver quality-of-life affects the level or quality of care that caregivers provide to their loved ones with AD. The dearth of research could be due to the absence of research questionnaires designed specifically to measure level or quality of care in AD. In the present study, we interviewed primary informal caregivers to obtain their views on the type of questionnaire that would be most suitable to assess level or quality of care in AD.

**Methods:**

A qualitative descriptive design was used. Purposive sampling was used to select participants. Participants were primary informal caregivers who were 18 years of age and older and were directly involved in the day-to-day care of community-dwelling (residing in private homes) persons with AD. A total of 21 caregivers were interviewed using focus groups or one-on-one interviews. Data were analyzed using qualitative content analysis.

**Results:**

Informal caregivers identified a number of factors that researchers should consider when developing an instrument to measure level or quality of care that informal caregivers provide to their loved ones with AD. Overall, caregivers preferred a questionnaire that would employ a case management approach that recognizes the increase in care demands as patient health deteriorates, that acknowledges the importance of social support for caregivers, and that considers the role of hired help.

**Conclusions:**

The information generated from this study can help in developing an instrument for measuring the level or quality of care provided. Such an instrument could guide nursing practice in supporting caregivers as they care for persons with AD.

## Background

Alzheimer’s disease (AD) is one of the leading causes of mortality and morbidity in North America. In 2013, approximately 450,000 people in the United States died as a result of Alzheimer’s disease [1]. Over 5 million Americans are presently living with the disease [2, 3]. Projections suggest up to 16 million Americans will have the disease by 2050 [3, 4].

In Canada, with a population of approximately 35 million people, more than 750,000 people are currently living with AD or other dementias [5, 6]. Further, approximately 40,000 Canadians develop the disease annually, with projections suggesting the total number of Canadians with AD or other dementias could double to 1.4 million people by 2030 [7, 8].

Globally, about 36 million people world-wide were living with AD in 2010 and this figure is set to top 115 million people by 2050 [6]. Further estimates suggest that AD will affect nearly 1 in every 85 people around the world over the next 40 years [9].

Primary or informal caregivers (usually spouses or children) provide the bulk of the care and support needed by their loved ones with AD. As opposed to formal caregivers, informal caregivers are not paid for the care they provide. AD caregivers perform a myriad of duties ranging from shopping for their loved ones’ groceries, helping with medications, managing finances and legal affairs, arranging medical care, and helping with basic and instrumental activities of daily living [7]. Caregiver duties vary depending on the clinical presentation of disease in their loved ones.

In the process of caring for their loved ones, caregivers often have to deal with multiple challenges, including their own personal well-being [10]. Studies have shown that the quality-of-life (QoL) experienced by unpaid caregivers of persons with AD is generally lower than the QoL of caregivers for persons who do not have AD [11]. The burden of caregiving in AD is tremendous and caregivers often experience high levels of stress and lower QoL [12]. Also, AD caregivers generally spend more time providing care than non-AD caregivers (for example, caregivers of persons with Parkinson’s disease) [13]. Findings from the Metlife Study of Alzheimer’s Disease suggest that 40 % of AD caregivers provide more than 40 hours a week of care, compared to only 28 % of caregivers for persons without AD [14]. As AD progresses, caregivers are called upon to perform an increasing range of tasks that ultimately include helping with basic activities such as bathing, toileting and dressing [10]. The burden associated with caring for someone with AD leads to decreased QoL among caregivers [11].

Caregiving also impacts health and many AD caregivers experience long-term deleterious effects on their general well-being, including increased risks for chronic disease, physiological changes (e.g. obesity, high blood pressure, and high cholesterol), higher health care utilization, major depression, and premature death [4]. In a meta-analysis to compare the physical health of AD family caregivers with demographically (e.g. age, sex, income, etc.) similar non-caregivers, the authors found that caregivers exhibited a greater risk for health problems than did non-caregivers [14]. Combined together, these factors result in early institutionalization of persons with AD [4, 15].

Research in other fields has suggested that decreased QoL could affect work productivity. Among adults aged 18 years or older who participated in the 2005 US National Health and Wellness Survey, lower QoL increased workplace absences and reduced job performance [16]. In the area of care provision to persons with AD, it is possible that these workplace productivity issues might translate into declining ‘caregiver productivity’.

We conducted a recent systematic review [17] and found that very little research has focused on the relationship between AD caregivers’ QoL and the level or quality of care they provide to persons with AD. The review identified only one study [18] that tangentially addressed the topic; the evidence was therefore insufficient to draw conclusions.

In that study [18], the variable that best approximated level of care was the total number of hours of care per day, either overall hours or the number of hours devoted to helping care recipients with Instrumental Activities of Daily Living (IADLs). The variables that best approximated quality of care included caregiver proficiency, as measured by the Caregiver Mastery Index (CMI) [18, 19], and caregiver skill enhancement, as measured by the Task Management Strategy Index (TMSI) [18]. The CMI is a six-item scale that uses a 5-point Likert format ranging from 1 (never) to 5 (always). A higher score means greater mastery of the caregiving role. Items on the CMI include questions such as “How often do you feel you should be doing more for the care recipient?”

The TMSI is a 19-item scale that measures the extent to which positive caregiving strategies are used to manage ADL dependence and problem behaviours in care recipients. The TMSI also employs a 5-point Likert format from 1 (never) to 5 (always). Higher scores on the TMSI indicate greater use of such strategies. Examples of items include the extent to which caregivers use strategies such as visual and tactile cueing or short instructions to communicate with their loved ones.

The CMI and TMSI may or may not be appropriate to measure AD caregivers’ level or quality of care. These instruments were designed to measure caregiver’s appraisal of his or her own ability to provide care and the extent to which positive caregiving strategies were used to manage problem behaviors respectively [18, 19].

One plausible explanation for the dearth of research on the relationship between QoL and level or quality of care is the lack of an instrument or questionnaire designed specifically to measure level or quality of care in AD [17]. We searched the literature for scales designed specifically to measure these constructs and found none. Most existing scales were designed to measure caregiver QoL or caregiver burden [20]. Literature searches in databases (Medline, PsycINFO, BIOSIS Previews, EconLit, Cochrane Reviews, and Social SciSearch) did not yield similar information for two other chronic, degenerative diseases (i.e., Parkinson’s disease [PD] and multiple sclerosis [MS]). The intent of looking at PD and MS was to see if any disease-specific literature might contain a discussion of caregiver QoL and the level or quality of care provided to care recipients. The search suggests that no research has been done to examine these relationships in these chronic diseases. Consequently, the development of a suitable questionnaire is a necessary prerequisite for conducting research in this important area.

To start the process of questionnaire development, we identified two questionnaires and sought caregiver input on whether and how these questionnaires could be modified to measure level or quality of care in AD. The first questionnaire was Macera et al.’s Caregiver Burden Scale (CBS) [21], which was originally designed to measure the perceived burden of AD caregivers. The CBS was one of the few instruments that included items relating to AD caregivers’ level or quality of provided care. The scale lists 15 domains in which persons with AD might require assistance, whether caregivers provide assistance in these domains, and whether the provision of this assistance adds to caregiver stress. The instrument’s developers defined perceived burden as the number of items for which: (a) the care recipient needed assistance, (b) the caregiver provided assistance and (c) the caregiver reported that providing assistance was stressful. Overall, this questionnaire captures most of the assistance that AD caregivers generally provide to their loved ones and it is more of a level of care scale since it captures the frequency at which caregivers perform selected duties [17, 18]. The instrument’s developers found the alpha coefficient for internal consistency for the CBS to be 0.87 [21].

The CBS was chosen for use in the current study because it was designed to be administered to AD caregivers and has some relevance to level of care provided (i.e. it lists tasks such as bathing and decision making that are performed by caregivers of persons with AD). The CBS also measures concepts related to quality of care, such as the type of care that caregivers provide to persons with AD and whether they administered medication to their loved one when they needed it [21, 22]. However, the CBS was not designed to measure level or quality of care; its purpose was to measure perceived caregiver burden [21]. Still, the content of this instrument is closely enough related to level and quality of care that it may serve as a template for developing a scale to measure level or quality of care.

The second instrument employed in this study was the Clinical Nurse Specialist (CNS) Performance Appraisal developed by Houston et al. [23]. This outcome-oriented performance appraisal tool was designed to measure CNS services by using eleven criteria with performance behaviors as agreed between clinical nurse specialists and their supervisors. The performance criteria considered by the CNS include enhancing clinical practice, education, consultation, research, and application of nursing theories in care provision. Responses are rated as 0 (“Does not meet performance criteria”), 10 (“standard or meets performance criteria”), or 20 (“above performance standard”) on each criterion. This instrument was selected for use in this study because it assesses performance using a scoring system that ranks the quality of care provided by individuals (e.g., meets criteria, exceeds criteria). Just like the CBS, the CNS serves as a source of components from which to build an instrument for measuring level or quality of care in AD.

The CBS and CNS were chosen for this study in part because of their inherent dissimilarity to one another. The hope in using such widely disparate instruments as a basis from which to start the focus groups and one-on-one interviews was to widen the basis of discussion and enrich participant feedback.

Results obtained from the present study will provide the information needed to develop a new instrument for measuring the level or quality of care provided by AD caregivers. Development of a new instrument will help researchers conduct studies to better understand how caregivers provide care to their loved ones with AD. Further, results from this study will help highlight the factors that are most important to AD caregivers as far as quality or level of care are concerned. This would help to guide nursing practice in ways to best support caregivers of persons with AD.

The following research question was addressed in this study:

What are caregiver perceptions of how the Caregiver Burden Scale (CBS) and Clinical Nurse Specialist (CNS) Performance Appraisal describe the level and quality of care that caregivers provide to their loved ones with Alzheimer’s disease?

## Methods

### Study design and sampling

A qualitative descriptive design was used. Purposive sampling was used to select participants for this study. Participants were primary informal caregivers who were 18 years of age or older, i.e., unpaid family members or friends who were directly involved in the day-to-day care of community-dwelling (residing in private homes) persons with AD [24–26]. We included males and females who self-identified as primary informal caregivers of a person with AD according to this definition, regardless of the disease severity of their loved ones. The caregiver sample represented a range of severity of illness of the loved ones with AD. We excluded caregivers who could not communicate in English.

Caregivers were recruited through the practices of three collaborating geriatricians in Hamilton and Toronto, Ontario. The geriatricians or their staff initially approached primary informal caregivers in their clinics to give them general information about the study and to ask for permission to be contacted by a member of the research team (AAH). Once caregivers agreed to be contacted, the geriatrician’s office forwarded their names to the research team for follow up. Caregivers were then mailed a letter to further explain the study and invite them to participate.

Caregivers who indicated an interest in participating were booked to attend a focus group or one-on-one interview, depending on their availability. Reminder letters were mailed to each caregiver one week before the scheduled interview to remind them about the day, time, and location of the interview.

A total of 21 caregivers participated in the study. Two focus group sessions were held with a total of twelve participants (six participants per group). The remaining nine caregivers participated in one-on-one interviews. Recruitment of additional caregivers ceased after these 21 participants because data saturation had been reached [25].

### Data collection

The approach of cognitive interviewing was used during the focus group and one-on-one interview sessions. In cognitive interviewing [27], researchers ask participants for their thoughts about a questionnaire. This includes what participants thought about the questions (e.g. were they easy to understand, did they mean for participants what the researchers thought they should mean, etc.), whether any content should be added or deleted from the questionnaires, and whether the format was pleasing to the eye. Researchers conducting a cognitive interview are not interested in testing the procedure for administering a questionnaire, nor are they interested in participants’ responses to the questions per se. Cognitive interviewing has many forms (e.g. think- aloud versus verbal probing) and can be used in both one-on-one and focus group interviews [28–31].

On the interview day, participants received an information package explaining the study. The interview facilitator (AAH) reviewed the package at the start of each session and obtained written informed consent after participants had a chance to ask questions about the study. Participation was voluntary and caregivers could withdraw at any time during a focus group or one-on-one interview.

For the focus groups, caregiver participants were asked to complete a brief demographic survey and the CBS and CNS questionnaires. Then, we obtained caregivers’ perceptions of the tools, as well as their suggestions for ways to revise the content of the tools. We intended for the suggested revisions to serve as a guide for the future development of an instrument to measure level and quality of care in AD. After completing the two questionnaires, participants were asked to reflect on the questions presented in each tool and to provide an opinion as to whether the questions adequately captured the complexity of their role as caregivers. Participants were asked to comment on how the questionnaires should be revised to become relevant for measuring level or quality of care in AD. All focus groups and one-on-one interviews were tape recorded and transcribed verbatim.

For the focus groups, the focus group facilitator (AAH) raised relevant questions for the group to discuss. Questions were open-ended queries (e.g., “*Please tell me about your experience completing these two questionnaires*” and “*Are there caregiver tasks or questions you think we should be asking that are not currently on the questionnaire?*”) designed to elicit discussions around the appropriateness of the two questionnaires for measuring level or quality of care. Clear group guidelines were issued at the start of the focus groups to encourage free-flowing discussion among participants. Each focus group lasted approximately two hours.

For participants who could not attend any of the scheduled focus group sessions, one-on-one interviews were conducted using a similar approach to the focus groups described above. The information obtained in one-on-one interviews is valuable because it represents individuals’ own unique positions and allows participants to express their views without the restrictions that the presence of other participants may impose. The data from these interviews were analyzed in the same manner as the focus groups [32, 33]. Each one-on-one interview lasted approximately one hour. The focus groups and one-on-one interviews took place between February 10, 2011 and October 15, 2012.

### Data analysis

Qualitative content analysis, consistent with a qualitative descriptive design, was the method of analysis for this study. Qualitative content analysis has been defined as a dynamic form of analysis of verbal data that is oriented toward summarizing the informational contents of that data [25]. One of the main advantages of this approach is that it helps group large amounts of text into manageable numbers of categories that denote similar meanings. Qualitative content analysis also provides a deep knowledge and understanding of the phenomenon under study [34, 35]. We used qualitative content analysis because our goal was to develop a robust understanding of the types of questions family caregivers would like to see in a questionnaire measuring level or quality of care from their perspective [36]. Consistent with this approach, the goal was to use content analysis in grouping the large amount of data obtained from the focus group and one-on-one interviews into meaningful categories or recommendations for questionnaire development.

Data were transcribed by a professional transcriber and the transcriptions were checked against the original recordings for accuracy. The primary author and data analyst (AAH) read all the interviews repeatedly to achieve immersion and gain a full understanding of the entire data obtained from caregivers during the interviews. The perusal of the interview transcripts was guided by the objectives of the study. Data were organized using NVIVO 10 (QSR International, Doncaster, Australia). Once a full understanding of the data was achieved, important words from each interview transcript that appeared to capture key thoughts or concepts were highlighted. The initial analysis commenced as the analyst (AAH) noted his initial thoughts and impressions about the data obtained from the focus group and one-on-one interviews. During this process, codes that represented groups of key thoughts among the participants were derived directly from the text, representing the initial coding scheme. Once the initial coding scheme was finalized, the codes were sorted based on how closely related they were to one another. Finally, the emergent categories from the data were grouped into meaningful clusters that represented the major findings from the study [37].

### Study rigour

Several strategies were used to ensure rigour. Memos were kept throughout the process of conducting this study. This included an audit trail of all decisions related to caregiver recruitment, data collection, analysis, and writing. Besides the principal author (AAH), other investigators (JP and MO) reviewed interview transcripts and provided feedback on the coding. Finally, the entire research team provided overall feedback on the analysis and study findings.

### Ethics approval

We obtained ethics approval from the McMaster University/Hamilton Health Sciences Research Ethics Board (REB#: 10–420), St. Joseph’s Healthcare Hamilton Research Ethics Board (REB#: 12–3706), and Sunnybrook Health Sciences Centre Research Ethics Board (REB#: 211–2011). All participants provided written, informed consent and understood that they could refuse to answer any questions or withdraw at any time.

## Results

### Caregiver sample

Table 1 presents the demographic profile of the 21 caregivers who participated in this study. These caregivers were typically older adults (Median age = 62.0 years; 25th percentile = 54.5 years; 75th percentile = 77.0 years) female (*n* = 18), technical/community college or university educated (*n* = 16), and the spouse or child of a person with AD (*n* = 20).

**Table 1:** Demographic characteristics of caregivers

Characteristics	Family caregivers
Age (years), median (25–75 percentile)	62 (53, 76)
Age range (years)	46–92
Gender, n (%)	
Women	18(86)
Men	3(14)
Education, n (%)	
Elementary school	2(10)
High school	3(14)
Technical/community college	8(38)
University	8(38)
Relationship to care recipient, n (%)	
Spouse	11(52.4)
Child	9 (42.8)
Other	1(4.8)
Current annual household income, n (%)	
$0–$30,000	2(9.5)
$30,001–$45,000	5(23.8)
$45,001–$60,000	6(28.6)
$60,000 or more	7(33.3)
Missing	1(4.8)

### Caregiver perceptions of the CBS and CNS scales

As a starting point for discussion, participants were asked to complete both the CBS and CNS. Neither scale is appropriate for measuring level or quality of care in AD, and the main purpose was to generate ideas on the components of a ‘good’ scale for measuring these constructs. The categories or themes identified in the interview transcripts that were related to caregiver perceptions of the instruments are: (a) use of a case management approach, (b) recognition of increasing care demands in relation to the declining status of the care recipient, (c) acknowledgment of the importance of social support for caregivers, and (d) consideration of the role of hired help.

### Use of a case management approach

In the context of the level or quality of care provided to their loved ones, caregivers reported preferring a questionnaire that had characteristics similar to the CBS scale. As unpaid family caregivers, they preferred a questionnaire that was relevant to their caregiving experience through the use of a case management approach. Case management is defined as an approach to level or quality of care that recognizes the central role that caregivers play in managing the continuum of care that persons with AD often require [38, 39]. According to caregivers, this approach to care aims to empower caregivers and facilitate timely access to essential care services to help support the needs of care recipients. Caregivers argued that since they typically perform multiple tasks (help with feeding, medication, financial planning, advocacy, etc.) at the same time, a questionnaire designed to measure level or quality of care must recognize both the diversity of their role and the inter-relationships between the various duties performed as part of their role. As stated in the following one-on-one interviews:

*Case management I guess is the way I’ll put it…I usually start off with all her medical needs. Then I take care of appointments, surgeries, pharmaceuticals, power of attorney,… I’m the primary contact for her…anything to do… finances, types of care, applying for her pills…You know I even got the [name of home care organization] involved… even taking her to church… (One-on-one interview #1)*

*I provide all those areas requiring assistance, transportation, housekeeping, cooking, shopping, decision making, financial keeping, walking, making home repairs, administering medication, dressing, bathing, toileting...., I have to do them all. As a matter of fact…I have virtually taken over full control and she’s virtually tethered to me… I think I am slowly approaching my inability to continue to do all these things for her because it’s almost becoming overwhelming and time to consider moving her into a nursing home. Not quite there yet but getting close (One-on-one interview #6).*

### Recognition of increasing care demands

Caregivers reported that they face unique and multiple challenges with their loved ones due to the ongoing decline exhibited in AD. Further, the needs of people with AD change from time to time and caregivers often find themselves performing progressively higher level of duties over time. Caregivers suggested that any questionnaire that is developed to assess the level or quality of care provided must consider the increasing care demands in relation to the declining status of the care recipient over time (for example, deteriorating behavioural health). Caregivers stated:

*Well, I mean if the caregiver gets a full nights' sleep, well that’s great…but if you are caring for somebody who is up wandering around the house all night, you’re not getting any sleep therefore the caregiver is tired the next day and could not give, you know, proper care to them… If you are rested you are bound to provide better care (One-on-one interview #7)*

*For me, I think it’s important to determine if there is going to be a time when your ability to provide the necessary level of care in each category is beyond your ability to do so (One-on-one interview #6)*

### Importance of social support

Further, participants indicated that they prefer a questionnaire that acknowledged the role of social support for family caregivers. Persons with AD often have a number of family caregivers who are jointly responsible for performing the duties being evaluated in a level or quality of care questionnaire. Caregiver support organizations such as the Alzheimer's Society also play a critical role by providing information and training to help caregivers provide an adequate level and quality of care. These organizations generate opportunities for caregivers to socialize and exchange ideas about caregiving practices. Many caregivers stated that the input of other family members (including neighbours) and the tips they pick up from socializing with other caregivers have great impact on the level or quality of care that they provide. These caregivers also believe that getting their loved ones involved in social activities is important for the level or quality of care provided. As one participant stated:

*(Social support) It’s very important, I think it’s important to involve the patient in outside activities, to involve them with family, you know, to get them out and around people as much as you can. I mean they are going to be at a point at some time when they won’t want to go out, when they won’t want to meet people. And I think you can probably, you know, stretch the time now that they’re still enjoying going out… We have friends that take my husband (person with AD) to coffee and take him out to football games, we all go to hockey games, participate in dinners and that sort of thing. So we try to keep him as social as possible… So, you need a question under social situations, you know, whether family are involved, sports activities, dining out. I know some people with Alzheimer’s are not comfortable dining out, their caregivers are not comfortable taking them to a restaurant (One-on-one interview #7).*

*I have a very dear friend and she is going through the same thing and we sort of talk about things together and we get annoyed at ourselves because we lose patience, but then it’s hard not to [right] you know. I think it’s very hard when a person is a clever person and a kind outgoing person to all of a sudden they can’t do those things because they just can’t remember. You know, and that to me is the hardest part, now I’m sure that’s up to each individual themselves [right] how they handle those (One-on-one interview #8).*

### Role of hired help

Caregivers also noted that the CBS and the CNS do not address the role of hired help or other care providers that families often pay to provide temporary reprieve for the regular caregiver. Hired help like those available through respite care provide temporary relief for the regular family caregiver to pursue other activities (for example, training or relationships with other caregivers) that may ultimately help improve the level or quality of care provided to the care recipient. Many participants suggested that a question about hired help should be incorporated into the questionnaire. The following interaction among participants attending a focus group highlights this criterion:

*P1: I spend money on additional caregiver help. It is much cheaper, and I am happy with my decision*

*P3: I agree with you…my mother needs these hook-ups from other sources…Just so they know they are not alone*

*P4: Support from other available sources is wonderful and much needed for me (Focus group #2)*

Other participants attending one-on-one interviews said:

*“He is a Veteran…through the Veterans he gets a chap that comes in every Monday and Thursday for two hours. And he will take him for a walk or take him out in the car for a little bit…and it’s good for me to take my garbage out and that sort of thing. Those things are very important and that means a lot to me.”(One-on-one interview #4)*

*When you get to a point where you simply cannot handle the person, respite care would probably be very good at some point so the caregiver could have a few days off, get away for a weekend (One-on-one interview #7)*

## Discussion

The current qualitative study was conducted to better understand AD caregiver perceptions of how we can best measure level or quality of care. By having caregivers complete the CBS and CNS scales, we gained valuable information that can be used to develop an instrument for measuring level or quality of care in AD. Although there was unanimous agreement about their preference for the CBS over the CNS when it comes to measuring care provided from an AD caregiver perspective, participants identified some limitations of the CBS. The main criticisms of this instrument stem from the fact that this scale, like the CNS, was not originally designed to measure level or quality of care. However, in the absence of an instrument designed specifically for this purpose, this instrument provides a solid foundation that can be improved upon to measure level or quality of care in AD. The CBS is simple, user-friendly, and contains questions about the everyday experience of AD caregivers. Although the CNS is comprehensive and covers many aspects of caregiving, caregivers perceive this questionnaire to be unconnected to the duties they actually perform on a day-to-day basis.

In response to questions about the lack of a questionnaire to measure level or quality of care provided to persons with AD, participants suggested that the CBS questionnaire should form the basis of an instrument to measure this construct. This instrument should include questions that address case management in caregiving, the increasing demands of care in relation to the declining status of care recipients, social support for caregivers, and the role of paid help to provide temporary relief for informal caregivers. Caregivers argued that the above categories all have important implications for the level or quality of care they provide to their loved ones. For example, friends and family (i.e. social support) may give a caregiver the encouragement needed to provide better quality of care for his or her loved one. Hiring a knowledgeable substitute carer (i.e. role of hired help) to provide a break for the primary caregiver could ultimately lead to higher level and quality of care for the care recipient.

Findings from this study reflected what has been found in other studies examining the needs of caregivers and care recipients in AD. In a recent study exploring the service and support needs of families with early-onset Alzheimer’s disease (EOAD), the authors identified the following three themes from their focus group interviews: (1) the challenges of providing care; (2) the challenges of accessing services and benefits; and (3) the desire for additional services tailored to meet the needs of caregivers and individuals with EOAD [40]. In a nationally representative survey describing both the use of and needs of 984 informal caregivers for people with dementia in the Netherlands, two thirds (67.4 %) of the caregivers indicated they had one or more needs for additional professional support. Among other support needs, informal caregivers stated that they often require help in deciding what to do when their loved one is frightened, angry, or confused [41].

In a study examining 25 physicians’ perspectives on care of persons with dementia, participants (physicians) in focus group discussions thought that much of the care received by persons with AD should come from support services such as government-run, community-based health and social service centres [42]. Although the physicians in the study admitted that they were not well informed about these services, they agreed that these alternate sources of care offer enormous opportunities for persons with AD and their family caregivers. These findings support the results from the current study identifying social support and role of hired help as important for the care that persons with AD receive.

However, engaging hired help has implications for the health economics of care for persons with AD. Although hiring outside help may result in a reduction in the burden or stress associated with caring for a person with AD, it may ultimately increase the financial burden of care [8]. Availability of government subsidies or other financial assistance may help reduce the financial burden on family caregivers as a result of hiring someone to care for their loved ones.

The above findings support the notion that family caregivers are better positioned to identify the needs of their loved ones with AD, and possess knowledge that can be utilized in meeting the needs of this population. The feedback provided by caregivers in the current study can be used to develop items for a questionnaire that measures level or quality of care in AD. The next step would be to use the feedback and develop a draft questionnaire and then ask AD caregivers whether it captures level/quality of care. A newly developed instrument would need to be validated among AD caregivers before it could be used in research. Examples of required validation would include an examination of test-retest reliability.

The current study has several implications for practice and research. Caregivers of persons with AD perform important functions, and the need for an instrument that is specifically designed for measuring the level or quality of care that these caregivers provide has been previously highlighted [17]. By developing an instrument for this purpose, it would be possible to rate the level or quality of care that AD caregivers provide to their loved ones. This understanding can be used to develop policies that provide support for caregivers. Results from this study could also help caregivers, caregiver support organizations, nurses and other healthcare professionals, and policymakers make informed decisions about programs to optimize the care that persons with AD receive from their caregivers. Examples of potential programs include enhanced respite care to relieve caregivers of the responsibility of providing all of the care required by their loved ones. This relief could help decrease caregiver stress and reinvigorate the level and quality of care that caregivers provide to their loved ones with AD. A new questionnaire developed for measuring level or quality of care could also be used to capture the impact of nursing and other programs on level or quality of care provided.

In summary, two vastly different scales were used in eliciting a range of opinions from caregivers regarding the measurement of level or quality of care in AD. Since both of these questionnaires were originally designed for a different purpose, it should be noted that neither of the two questionnaires is really appropriate for measuring level or quality of care in AD. However, the simplicity of the CBS, as well as the fact that it was designed to be used by AD caregivers, makes it possible to borrow some of its components to develop a new questionnaire that incorporates the findings from this study.

## Strengths and limitations

The use of both focus group and one-on-one interviews is a major strength of this study. While focus groups draw on group interplay, one-on-one interviews rely on individual perspectives. Triangulation of one-on-one and focus group interview data helped enhance the credibility of the findings by enabling cross verification from these two forms of interview. Also, the use of two dissimilar questionnaires as a starting point for discussion gave participants a prompt from which to express a wide range of opinions during the focus groups or interviews. Readers should note that the caregiver sample was recruited from geriatric practices in Southern Ontario, Canada. The views expressed by this sample might differ from the opinions of caregivers who are recruited from other settings. There may be biases related to the use of pre-specified instruments as basis for discussion in this study. Participants’ responses may vary if other relevant instruments had been chosen or if caregivers had been asked to comment in general about the care they provide without the use of pre-selected instruments. Further, the caregivers interviewed in this study were all caring for people with AD. The findings may not be generalizable to caregivers of people with other types of dementia (for example, early-onset frontotemporal or Lewy body dementia).

## Conclusion

The information generated from this study can help in developing an instrument for measuring the level or quality of care that AD caregivers provide to their loved ones with AD. Such an instrument could be useful in nursing practice to help identify caregiver needs and guide interventions to help caregivers provide optimal care.

## Abbreviations

AD: Alzheimer’s disease
ADL: Activities of Daily Living
CBS: Caregiver Burden Scale
CMI: Caregiver Mastery Index
CNS: Clinical Nurse Specialist Performance Appraisal
EOAD: Early-onset Alzheimer’s disease
IADLs: Instrumental Activities of Daily Living
QoL: Quality-of-life
REB: Research Ethics Board
TMSI: Task Management Strategy Index.
